# Dataset on bryophyte species distribution across an elevational gradient on Flores Island

**DOI:** 10.3897/BDJ.13.e165664

**Published:** 2025-11-19

**Authors:** Rosalina Gabriel, Leila N. Morgado, Silvia Poponessi, Débora S. G. Henriques, Márcia C. M. Coelho, Gabriela M. Silveira, Fernando Pereira, Paulo A.V. Borges

**Affiliations:** 1 University of Azores, CE3C—Centre for Ecology, Evolution and Environmental Changes, Azorean Biodiversity Group, CHANGE —Global Change and Sustainability Institute, School of Agricultural and Environmental Sciences, Rua Capitão João d’Ávila, Pico da Urze, 9700-042, Angra do Heroísmo, Azores, Portugal University of Azores, CE3C—Centre for Ecology, Evolution and Environmental Changes, Azorean Biodiversity Group, CHANGE —Global Change and Sustainability Institute, School of Agricultural and Environmental Sciences, Rua Capitão João d’Ávila, Pico da Urze, 9700-042 Angra do Heroísmo, Azores Portugal; 2 Institute for Alpine Environment, Eurac Research, Viale Druso 1, 39100, Bolzano, Italy Institute for Alpine Environment, Eurac Research, Viale Druso 1, 39100 Bolzano Italy; 3 Banco Genético Vegetal Autóctone, Empresa Municipal Cascais Ambiente, Estrada de Vale de Cavalos, 2645-138, Cascais, Portugal Banco Genético Vegetal Autóctone, Empresa Municipal Cascais Ambiente, Estrada de Vale de Cavalos, 2645-138 Cascais Portugal; 4 University of the Azores, Gaspar Frutuoso Foundation, Rua Capitão João D'Ávila, Pico da Urze, São Pedro, 9700-042, Angra do Heroísmo, Portugal University of the Azores, Gaspar Frutuoso Foundation, Rua Capitão João D'Ávila, Pico da Urze, São Pedro, 9700-042 Angra do Heroísmo Portugal; 5 IUCN SSC Monitoring Specialist Group, Angra do Heroísmo, Azores, Portugal IUCN SSC Monitoring Specialist Group Angra do Heroísmo, Azores Portugal

**Keywords:** Azores, bryoflora, elevational gradient, Flores Island, GIMS protocol, liverworts, mosses, MOVECLIM-AZO, natural park, substrates

## Abstract

**Background:**

A bryophyte diversity survey was carried out from July 29 to 1 August 2013, in Santa Cruz das Flores, Flores Island (Azores) (39.471185 N Latitude; -31.184692 W Longitude), along an elevational gradient (70, 200, 400, 600 and 800 m a.s.l.). The study employed the Global Island Monitoring Scheme (GIMS) protocol for bryophytes. At each elevation level, three replicates of six substrates colonised by bryophytes (rock, soil, humus, dead wood, tree trunks, leaves) were collected. In total, 385 sampling events generated 1345 species occurrence records, representing 89 bryophyte species (37 mosses; 52 liverworts).

**New information:**

The acrocarpous moss *Fissidens azoricus* (P.de la Varde) Bizot, listed as "Critically Endangered" by the IUCN, had not been reported from Flores Island since 1937; this is the first publication with new locations for the species.

The altitudinal gradient revealed an increase in species richness and abundance with elevation, following a mid-elevation peak pattern, with the highest richness between 400 m a.s.l. (52 species; 364 records) and 600 m a.s.l. (54 species; 402 records), followed by a decline at 800 m a.s.l. (33 species; 148 records). At 70 m a.s.l., 20 species were identified (128 records) and, at 200 m a.s.l., 35 species were recorded (232 records). In terms of substrate preference, the highest species richness and abundance were found colonising epiphytic substrates (58 species; 424 records), followed by terricolous substrates (44 species; 233 records).

## Introduction

Understanding elevational diversity patterns is essential for guiding conservation actions, predicting the potential community shifts under environmental change scenarios and identifying priority areas for biodiversity protection. Previous studies conducted in the Azores Archipelago have reported high bryophyte species richness on both Pico (Coelho et al. 2021, Gabriel et al. 2024b) and Terceira Islands (Henriques et al. 2017, Coelho et al. 2021, Gabriel et al. 2024a) with richness peaking between 600 and 1000 m a.s.l. This pattern has been largely attributed to the presence of relatively undisturbed native forests at these elevations (Coelho et al. 2016, Henriques et al. 2017, Gabriel et al. 2024a, Gabriel et al. 2024b). In contrast, Flores Island, the westernmost in the Archipelago, had not been intensively surveyed for over two decades. A compilation of literature referencing bryophyte records from Flores Island includes 85 references published between 1844 and 2023, encompassing journal articles, books and book chapters. Notable contributions include Erik Sjögren, Cecília Sérgio, Pierre and Valentine Allorge and Manuela Sim-Sim (see Suppl. material 1).This data paper aims to address the existing knowledge gap concerning bryophyte diversity on Flores Island.

## General description

### Purpose

The main objectives of this study were:

1. to conduct a comprehensive survey of bryophytes within the Flores Natural Park, employing the Global Island Monitoring Scheme (GIMS) protocol (Gabriel et al. 2014, Borges et al. 2018), along a transect spanning an elevational gradient from 70 to 800 m a.s.l.;
2. to compile the publications referencing bryophyte records from Flores Island.

## Project description

### Title

Inventory of bryoflora present in different altitudinal gradients and substrates of Flores Island (Azores)

### Personnel

Project conceptualisation: The MOVECLIM project, "Montane vegetation as listening posts for climate change", was originally initiated by the research team led by Claudine Ah-Peng to study and promote tropical bryophytes and ferns as bioindicators of climate change. The project was coordinated in the Azores by Rosalina Gabriel. The monitoring protocol implemented in the project was the GIMS protocol (Ah-Peng et al. 2012, Borges et al. 2018, Gabriel et al. 2024a). This is a standardised method that has also been applied in tropical settings, including La Réunion (Ah-Peng et al. 2012), Madagascar (e.g. Marline et al. (2020)) and other locations.

FIELDWORK:

Site selection and experimental setting: Rosalina Gabriel and Fernando Pereira.

Permits: The Azores Government, through the Environment Department, gave the necessary authorisations to work within the Flores Natural Park. Sample collection: The bryoflora inventory was conducted between 29 July and 1 August 2013, within the Municipality of Santa Cruz das Flores. Sampling was carried out at six sites differing in location, elevation (m a.s.l.) and geographic coordinates (decimal Latitude/Longitude). The fieldwork was coordinated by Rosalina Gabriel, with the participation of Márcia C. M. Coelho, Débora S. G. Henriques and Fernando Pereira.

LAB WORK:

Bryophyte Taxonomic Work: The taxonomic identification of the bryophyte specimens was primarily conducted by Silvia Poponessi, with contributions from Margarida Brito de Azevedo and Rosalina Gabriel. Between 2024 and 2025, a selected subset of specimens was reviewed by Leila N. Morgado and Gabriela M. Silveira, under the supervision of Rosalina Gabriel. Manuela Sim-Sim and Cecília Sérgio helped with the identification of particularly challenging specimens.

MANAGEMENT:

Voucher specimen management: Márcia C. M. Coelho, Débora S. G. Henriques, Rosalina Gabriel.

Database management: Rosalina Gabriel.

Darwin Core databases: Leila N. Morgado, Paulo A. V. Borges, Rosalina Gabriel.

### Study area description

Flores Island, one of the nine islands of the Azores Archipelago (Portugal), is situated west of the Mid-Atlantic Ridge, resting on the North American lithospheric plate (Forjaz 2014). Flores is the fourth smallest and the fourth lowest island of the Archipelago, with a land area of 143 km² and a highest elevation of 911 m (Morro Alto). Remnants of well-preserved native vegetation persist, predominantly in the form of Juniperus brevifolia forests (Elias et al. 2016) and mire ecosystems (Dias and Melo 2010). While montane forests in the Azores generally occur at elevations between 700 and 1000 m a.s.l., on Flores, they are found at lower altitudes. This shift is attributed to the island’s high precipitation levels and the presence of Sphagnum spp. (Bryophyta, Sphagnaceae) dominated soils, which provide favourable conditions for the development and maintenance of these forest communities (Elias et al. 2016).

### Design description

The bryophyte inventory in the field was conducted from 29 July to 1 August 2013, under the supervision of RG, with field contributions from authors DSGH, MCMC and FP. This fieldwork follows the GIMS protocol for bryophyte sampling (Borges et al. 2018), incorporating both taxonomic information on bryophytes (Ah-Peng et al. 2012) and a suite of environmental variables (Gabriel et al. 2014). Five sites were selected along an altitudinal gradient, spaced at 200-m intervals between 70 m and 800 m above sea level. At each site, two 10 × 10-m plots were established within areas of uniform vegetation structure, with a distance of 10 to 15 m between them. Each plot was subdivided into 25 quadrats (2 m x 2 m), from which three were randomly selected for detailed analysis. From each quadrat, three microplots of 5 cm × 10 cm were collected across six substrate types: rock (rupicolous), soil (terricolous), organic matter (humicolous), decaying wood (lignicolous), bark (epiphytic, at three vertical strata) and leaf/frond surfaces (epiphyllous), whenever possible. Microplots from tree bark were collected in a stratified manner across three vertical zones: low (1–50 cm), medium (51–100 cm) and high (101–200 cm). Specimens were identified in the laboratory to the lowest possible taxonomic level. Additionally, data on cover-abundance and sociability were estimated for each bryophyte taxon within every microplot.

### Funding

This study was originally funded by the ERANET BIOME MOVECLIM project "Montane vegetation as listening posts for climate change" (Regional Government of the Azores, grant M2.1.2/F/04/2011/NET). MCMC was supported by the Regional Government of the Azores through FRCT (grant M3.1.2/F/007/2012). RG and PAVB received support from the Azores DRCT Pluriannual Funding (M1.1.A/FUNC.UI&D/010/2021-2024) and FCT (UIDB/00329/2020-2024; DOI: 10.54499/UIDB/00329/2020), under Thematic Line 1 – Integrated ecological assessment of environmental change on biodiversity. This study is part of the Biodiversa+ project BioMonI – Biodiversity monitoring of island ecosystems, funded by FCT (BiodivMon/0003/2022), which also supported Open Access and provided funding to LNM, RG and PAVB.

## Sampling methods

### Study extent

Following the protocol explained above, under 'Design description', each site comprised two 100 m² plots, spaced at 200 m a.s.l., ranging from 70 to 800 m above sea level. The elevational transect extended from Ponta do Ilhéu (70 m a.s.l.) to Morro Alto (800 m a.s.l.). The sampling location names and their corresponding coordinates are listed in Table 1 and illustrated in Fig. 2. At each plot, a general survey of the vascular plant species was performed, noting also the inclination and exposition of the areas and other relevant descriptive details.

### Sampling description

First, each quadrat was carefully observed to check the availability of substrates colonised with bryophytes. Next, microplots of 5 cm x 10 cm, placed on three replicates of the same substrate, were collected with a knife or tweezers to paper bags, identified with the name of the site, altitude, plot, quadrat, substrate and number of the replicate. Other data, such as the phorophyte name and information on evaporation, light, humidity and rugosity of the substrate were also collected.

### Quality control

FIELD SAMPLING: Sampling plots were established in environmentally homogeneous zones representing the most characteristic native vegetation at each surveyed elevation. All collections were conducted by bryologists, who ensured methodological consistency and minimised disturbance by avoiding excessive removal of bryophyte material. PREPARATION AND STORAGE: Microplot samples were placed in paper bags and left open in a darkened room until thoroughly dehydrated. Once identification was completed, each sample was transferred to labelled herbarium envelopes. These envelopes are deposited in the Bryophyte Section of the Herbarium of the University of the Azores (AZU), under the designation MOVECLIM – AZORES Project: Bryophytes from Flores Island (2013). SPECIES IDENTIFICATION (TAXONOMY): Comprehensive efforts were undertaken to ensure precise taxonomic identification:

1. the most current taxonomic keys and floras were employed under the guidance or direct supervision of experienced bryologists;
2. problematic specimens were submitted to specialists for further assessment and confirmation; and
3. specimens that were extremely small or etiolated were not identified to the species level.

Moss specimens were identified using the floras of Smith (2004) and Casas et al. (2006), while liverworts were determined based on the works of Paton (1999) and Casas et al. (2009) and the taxonomic key provided by Schumacker and Vána (2005). Identification was further supported by visual references, including Atherton et al. (2010), Lüth (2019) and Elias et al. (2022), alongside digital resources from the British Bryological Society and the Bildatlas der Moose Deutschlands. Nomenclature adhered to Gabriel et al. (2010) and Gabriel et al. (2011) incorporating updates available through the PBA (2025). DATA REGISTRATION: All identification and metadata were systematically recorded in a structured database in an Excel file. HERBARIUM DEPOSITION: Voucher specimens were deposited in the Bryophyte Section of the Herbarium of the University of the Azores (AZU-B), with proper labelling and long-term conservation measures. DATA STANDARDISATION AND DISSEMINATION: To ensure data accessibility, these records were formatted following the Darwin Core Archive (DwC-A) standard, facilitating integration into the Global Biodiversity Information Facility (GBIF) and other biodiversity platforms. A core event table was generated, comprising 385 sampling event records, each event corresponding to a single 5 cm × 10 cm microplot. An occurrence extension table was created, including 1345 records that detail all bryophyte taxa identified within the sampled microplots. The standardised data package was published through GBIF and associated biodiversity repositories, contributing to global efforts in bryophyte documentation and conservation research.

## Geographic coverage

### Description

**Figure 1.**
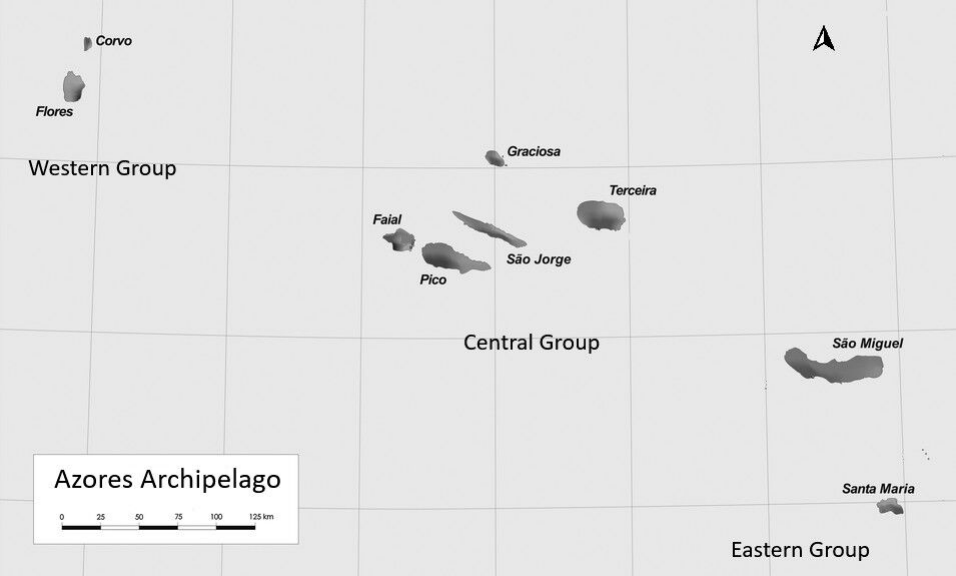
Map illustrating the grouping of islands in the Azores Archipelago, with Flores Island in the western group.

**Figure 2.**
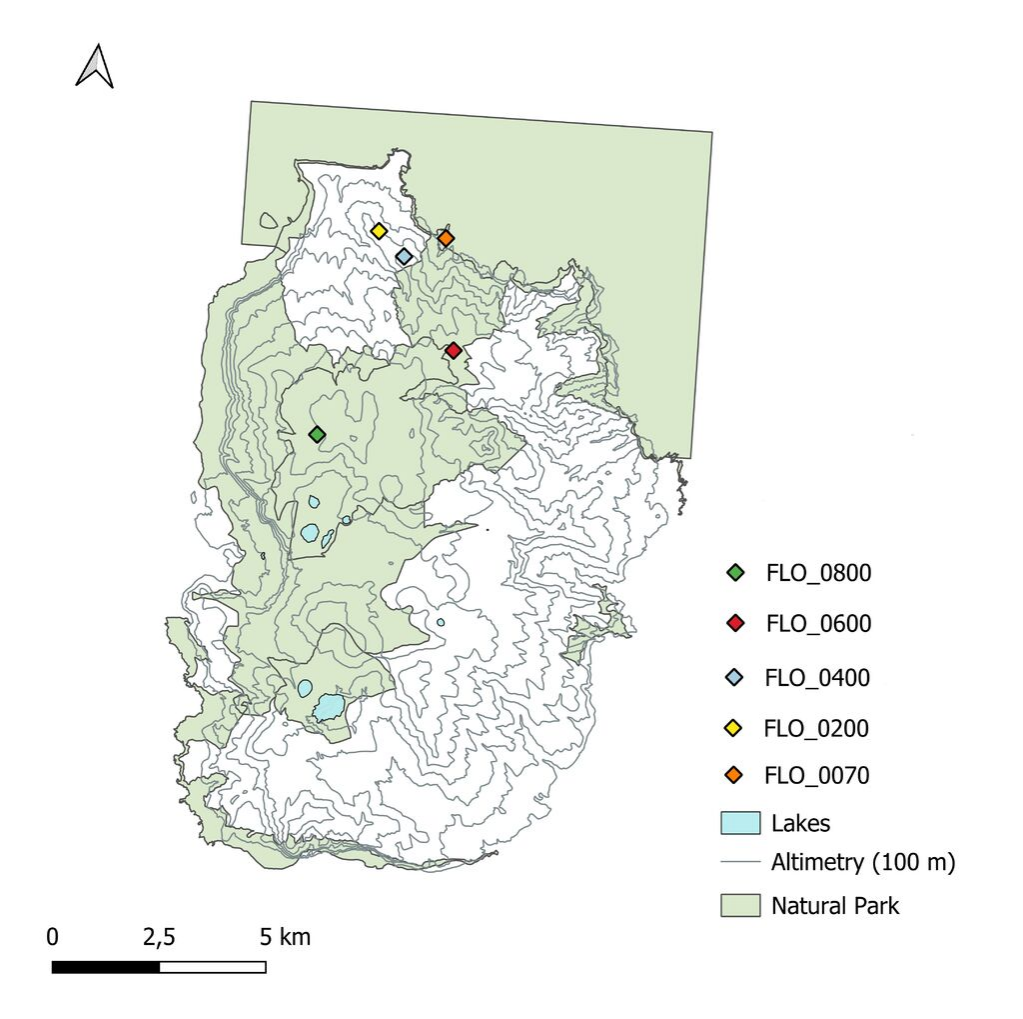
Map of Flores Island showing the bryophyte sampling points surveyed in 2013, in relation to the island’s Natural Park boundaries, altimetry and main lakes.

The research was carried out on Flores Island, one of the nine islands comprising the Azores Archipelago (Portugal) (Fig. 1). Of the five sampled points, three are located within the limits of the Flores Natural Park, in the Municipality of Santa Cruz das Flores (Fig. 2).

### Coordinates

39.5420 and 39.3470 Latitude; -31.3170 and -31.1020 Longitude.

## Taxonomic coverage

### Description

The bryophyte specimens collected in this survey comprise representatives of the phyla Bryophyta (mosses) and Marchantiophyta (liverworts). No representatives of the phylum Anthocerotophyta (hornworts) were recorded.

## Temporal coverage

### Notes

The sampling was performed from 29 July to 1 August 2013.

## Collection data

### Collection name

MOVECLIM-AZO-FLO_2013_Bryophytes from Flores Island

### Collection identifier

76349556-a70a-4ecc-88a7-cd085b6c875d

### Parent collection identifier

AZU_Section Bryophytes

### Specimen preservation method

Herbarium preservation

### Curatorial unit

Herbarium packet

## Usage licence

### Usage licence

Creative Commons Public Domain Waiver (CC-Zero)

### IP rights notes

Additional data or information related to this study can be obtained by contacting the corresponding author.

## Data resources

### Data package title

The MOVECLIM – AZORES project: Bryophytes from Flores Island (2013).

### Resource link

http://ipt.gbif.pt/ipt/resource?r=moveclim_azores_flores_island_2012

### Alternative identifiers

https://www.gbif.org/dataset/f846b120-a1a7-4b1d-a4b5-e0baf6f46775

### Number of data sets

2

### Data set 1.

#### Data set name

Event Table

#### Data format

Darwin Core Archive

#### Character set

UTF-8

#### Download URL

http://ipt.gbif.pt/ipt/resource?r=moveclim_azores_flores_island_2012

#### Data format version

V1.1

#### Description

The dataset was published in the Global Biodiversity Information Facility platform, GBIF (Gabriel et al. 2025). The following data table includes all the records for which a taxonomic identification of the species was possible. The dataset submitted to GBIF is structured as a sample event dataset that has been published as a Darwin Core Archive (DwCA), which is a standardised format for sharing biodiversity data as a set of one or more data tables. The core data file contains 385 records (eventID). This GBIF IPT (Integrated Publishing Toolkit, Version 2.5.6) archives the data and, thus, serves as the data repository. The data and resource metadata are available for download in the Portuguese GBIF Portal IPT (Gabriel et al. 2025).

**Data set 1.** 

Column label	Column description
eventID	Identifier of the events, unique for the dataset.
type	Type of the record, as defined by the Dublin Core Standard.
datasetName	Name of the dataset that in current projects is "MOVECLIM-AZO_2013_Bryophytes from Flores Island".
samplingProtocol	The sampling protocol used to collect the species.
eventDate	Range during which the record was collected.
day	The day of the month on which the Event occurred.
month	The month in which the Event occurred.
year	The year in which the Event occurred.
habitat	The habitat for an Event.
continent	The name of the continent in which the Location occurs (Europe).
islandGroup	Name of archipelago, always Azores in the dataset.
island	Name of the island, always Flores in the dataset.
country	Country of the sampling site, always Portugal in the dataset.
countryCode	ISO code of the country of the sampling site, always PT in the dataset.
municipality	Municipality of the sampling site, always Santa Cruz das Flores in the dataset.
locality	The specific name of the locality.
verbatimElevation	The original description of the elevation (altitude above sea level in metres) of the location.
verbatimCoordinates	Original coordinates recorded.
decimalLatitude	Approximate decimal latitude.
decimalLongitude	Approximate decimal longitude.
geodeticDatum	Standard Global Positioning System coordinate reference for the location of the sample collection points.
coordinateUncertaintyInMetres	Uncertain value of coordinate metrics.
coordinatePrecision	Value in decimal degrees to a precision of five decimal places.
georeferenceSources	Navigation system used to record the location of sample collections.

### Data set 2.

#### Data set name

Occurrence table

#### Data format

Darwin Core Archive format

#### Character set

UTF-8

#### Download URL

http://ipt.gbif.pt/ipt/resource?r=moveclim_azores_flores_island_2012

#### Data format version

V1.1

#### Description

The dataset was published in the Global Biodiversity Information Facility platform, GBIF (Gabriel et al. 2025). The following data table includes all the records for which a taxonomic identification of the species was possible. The dataset submitted to GBIF is structured as an occurrence table that has been published as a Darwin Core Archive (DwCA), which is a standardised format for sharing biodiversity data as a set of one or more data tables. The core data file contains 1345 records (occurrenceID). This GBIF IPT (Integrated Publishing Toolkit, Version 2.5.6) archives the data and, thus, serves as the data repository. The data and resource metadata are available for download in the Portuguese GBIF Portal IPT (Gabriel et al. 2025).

**Data set 2.** 

Column label	Column description
eventID	Identifier of the events, unique for the dataset.
licence	Reference to the licence under which the record is published.
institutionID	The identity of the institution publishing the data.
institutionCode	The code of the institution publishing the data.
collectionID	Identifier of the collection, unique for each specimens are conserved.
collectionCode	The code of the collection where the specimens are conserved.
datasetName	Project reference.
type	Characteristics of the object of study.
basisOfRecord	The nature of the data record.
dynamicProperties	A list of additional measurements, facts, characteristics or assertions about the record, including IUCN categories and colonisation status of taxa.
occurrenceID	Identifier of the record, coded as a global unique identifier.
recordNumber	An identifier given to the Occurrence at the time it was recorded.
recordedBy	A list (concatenated and separated) of names of people, groups or organisations responsible for recording the original Occurrence.
identifiedBy	A list (concatenated and separated) of names of people, who made the identification.
dateIdentified	Date of species identification.
disposition	The current state of a specimen with respect to the collection identified in collectionCode or collectionID.
taxonRank	Lowest taxonomic rank of the record.
kingdom	Kingdom name.
phylum	Phylum name.
class	Class name.
order	Order name.
family	Family name.
genus	Genus name.
specificEpithet	Specific epithet.
infraspecificEpithet	Infraspecific epithet at subspecies level.
scientificNameAuthorship	The authorship information for the scientificName formatted according to the conventions of the applicable nomenclaturalCode.
ScientificName	Complete scientific name including author.
organismQuantity	A number or enumeration value for the quantity of organisms (i, solitary specimen - one or few individuals; p, occasional and less than 5% cover; 1, less than 5% cover of total area; 2, 5%-25% of total area; 3, 25%-50% of total area; 4, 50%-75% of total area; 5, 75%-100% of total area).
organismQuantityType	Braun-Blanquet Scale.
establishmentMeans	The process of establishment of the species in the location, using a controlled vocabulary: 'Azores endemic', 'European endemic', 'Macaronesian endemic', 'native'.
occurrenceRemarks	Remarks on the material or surface where the biological specimen was collected.

## Additional information

This inventory of the bryoflora of Flores Island (Azores) recorded 385 sampling events, yielding 1345 identified specimens, representing 37 families, 58 genera and 89 species, with a average number of species per sample of 3.26.

Of all the specimens collected, only 5.4% could not be identified to species level, a relatively low proportion, indicating a high level of taxonomic resolution.

The phylum Marchantiophyta (liverworts) was the most represented, accounting for 52 species (58.43%), followed by phyllum Bryophyta (mosses) with 37 species (41.57%). No representatives of the phylum Anthocerotophyta (hornworts) were collected during this study.

In terms of colonisation status, most species were classified as native (n = 66). Additionally, 23 endemic species were identified, with varying geographic ranges: 12 are considered European endemics, seven are Macaronesian endemics and four are endemic to the Azores. No invasive bryophytes were found within the sampled sites.

According to the IUCN Red List (Hodgetts et al. 2019), one species is considered Critically Endangered (*Fissidens azoricus*), four species are considered Endangered (*Echinodium renauldii*, *Calypogeia azorica*, *Cololejeunea sintenisii*, Leptoscyphus porphyrius subsp. azoricus), another four species are considered Vulnerable (*Isothecium prolixum*, *Andoa berthelotiana*, *Cololejeunea azorica*, *Lejeunea mandonii*), 14 species are considered Near Threatened, while 64 species are classified as Least Concern (Table 2). Two species are not evaluated: *Daltonia lindigiana*, an American moss species restricted to the Azores in Europe and *Hypnum resupinatum*, sometimes considered part of the *Hypnum cupressiforme* complex (Hodgetts et al. 2020).

Additionally, two Azorean endemics and one Macaronesian endemic are classified as Endangered and two are listed as Vulnerable, according to the IUCN Red List (Table 2). Notably, this study recorded the presence of *Fissidens azoricus*, a species listed as Critically Endangered by the IUCN and not recorded on Flores Island since 1937. This emphasises the importance of bryophyte inventory efforts for conservation and biodiversity monitoring.

## Supplementary Material

10CED92F-7AE2-5E79-BA38-B0D9A7987A58 10.3897/BDJ.13.e165664.suppl1 Supplementary material 1 Compilation of Publications Data type Compilation of Publications Brief description List of Publications on Bryophytes in Flores Island (Azores) from 1844 to 2023. File: oo_1432716.csv https://binary.pensoft.net/file/1432716 Rosalina Gabriel

## Figures and Tables

**Table 1.** Bryophyte sampling sites of the MOVECLIM Project on Flores Island (Azores), surveyed in 2013. The table includes plot codes, locality names, elevations (m a.s.l.), slope, exposure and geographic coordinates (decimal degrees).

Plot Code	Locality	Elevation (m a.s.l.)	Slope	Exposure	Latitude	Longitude
TER_0070_P1	Ponta do Ilhéu	70	15	280	39.50633	-31.19453
TER_0070_P2		77	18	107	39.50622	-31.19461
TER_0200_P1	Caminho para Ponta Delgada	249	35	141	39.50689	-31.21286
TER_0200_P2		266	32	14	39.50661	-31.21275
TER_0400_P1	Outeiros	399	40	81	39.50192	-31.20558
TER_0400_P2		399	41	54	39.50183	-31.20558
TER_0600_P1	Ribeira do Cascalho	649	5	90	39.48281	-31.19042
TER_0600_P2		648	8	176	39.48267	-31.19033
TER_0800_P1	Morro Alto	833	14	278	39.46319	-31.22594
TER_0800_P2		833	15	283	39.46325	-31.22600

**Table 2.** List of species and subspecies sampled on Flores Island, with their colonisation status categories (Azorean endemic, European endemic, Macaronesian endemic and Native) and IUCN status (Critically Endangered, Endangered, Least Concern, Near Threatened, Vulnerable and Not Evaluated).

Phylum	Scientific Name	Colonisation status	IUCN Status
Bryophyta	*Andoa berthelotiana* (Mont.) Ochyra	Macaronesian endemic	Vulnerable
	*Atrichum undulatum* (Hedw.) P.Beauv.	Native	Least concern
	*Brachythecium mildeanum* (Schimp.) Schimp.	Native	Least concern
	*Campylopus flexuosus* (Hedw.) Brid.	Native	Least concern
	*Campylopus shawii* Wilson	Native	Least concern
	*Daltonia lindigiana* Hampe	Native	Not evaluated
	*Echinodium renauldii* (Cardot) Broth.	Azorean endemic	Endangered
	*Exsertotheca crispa* (Hedw.) S.Olsson, Enroth & D.Quandt	Native	Least concern
	*Fissidens adianthoides* Hedw.	Native	Least concern
	*Fissidens azoricus* (P.de la Varde) Bizot	Azorean endemic	Critically endangered
	*Fissidens crispus* Mont.	Native	Least concern
	*Fissidens polyphyllus* Wilson ex Bruch & Schimp.	Native	Least concern
	*Fissidens serrulatus* Brid.	Native	Least concern
	*Fissidens taxifolius* Hedw.	Native	Least concern
	*Heterocladium flaccidum* (Schimp.) A.J.E.Sm.	Native	Least concern
	*Heterocladium wulfsbergii* I.Hagen	Native	Least concern
	*Hypnum jutlandicum* Holmen & E.Warncke	Native	Least concern
	*Hypnum resupinatum* Taylor	Native	Not evaluated
	*Hypnum uncinulatum* Jur.	European endemic	Least concern
	*Isopterygiopsis pulchella* (Hedw.) Z.Iwats.	Native	Near threatened
	*Isothecium prolixum* (Mitt.) M.Stech, Sim-Sim, Tangney & D.Quandt	Macaronesian endemic	Vulnerable
	*Kindbergia praelonga* (Hedw.) Ochyra	Native	Least concern
	*Leucobryum glaucum* (Hedw.) Ångstr.	Native	Least concern
	*Leucobryum juniperoideum* (Brid.) Müll.Hal.	Native	Least concern
	*Mnium hornum* Hedw.	Native	Least concern
	*Nogopterium gracile* (Hedw.) Crosby & W.R.Buck	Native	Least concern
	*Polytrichum commune* Hedw.	Native	Least concern
	*Pseudoamblystegium subtile* (Hedw.) Vanderp. & Hedenäs	Native	Least concern
	*Pseudotaxiphyllum laetevirens* (Dixon & Luisier ex F.Koppe & Düll) Hedenäs	European endemic	Near threatened
	*Rhynchostegiella azorica* Hedenäs & Vanderp.	Azorean endemic	Near threatened
	*Sematophyllum substrumulosum* (Hampe) E.Britton	Native	Least concern
	*Sphagnum palustre* L.	Native	Least concern
	*Sphagnum papillosum* Lindb.	Native	Least concern
	Tetrastichium virens (Cardot) S.P.Churchill	Macaronesian endemic	Near threatened
	*Thamnobryum maderense* (Kindb.) Hedenäs	Native	Near threatened
	*Thuidium tamariscinum* (Hedw.) Schimp.	Native	Least concern
	*Trichostomum brachydontium* Bruch	Native	Least concern
Marchantiophyta	*Calypogeia arguta* Nees & Mont.	Native	Least concern
	*Calypogeia azorica* Bischl.	Macaronesian endemic	Endangered
	*Calypogeia fissa* (L.) Raddi	Native	Least concern
	*Calypogeia muellerian*a (Schiffn.) Müll.Frib.	Native	Least concern
	*Cephalozia bicuspidata* (L.) Dumort.	Native	Least concern
	*Chiloscyphus polyanthos* (L.) Corda	Native	Least concern
	*Cololejeunea azorica* V.Allorge & Jovet-Ast	Native	Vulnerable
	*Cololejeunea microscopica* (Taylor) Schiffn.	Native	Least concern
	*Cololejeunea sintenisii* (Steph.) Pócs	Native	Endangered
	*Colura calyptrifolia* (Hook.) Dumort.	Native	Least concern
	*Drepanolejeunea hamatifolia* (Hook.) Schiffn.	Native	Least concern
	*Frullania acicularis* Hentschel & von Konrat	Native	Near threatened
	*Frullania azorica* Sim-Sim, Sérgio, Mues & Kraut	European endemic	Least concern
	*Frullania microphylla* (Gottsche) Pearson	European endemic	Least concern
	*Frullania teneriffae* (F.Weber) Nees	European endemic	Least concern
	*Fuscocephaloziopsis crassifolia* (Lindenb. & Gottsche) Váňa & L.Söderstr.	Native	Least concern
	*Fuscocephaloziopsis lunulifolia* (Dumort.) Váňa & L.Söderstr.	Native	Least concern
	*Geocalyx graveolens* (Schrad.) Nees	Native	Near threatened
	*Harpalejeunea molleri* (Steph.) Grolle	Native	Least concern
	*Heteroscyphus denticulatus* (Mitt.) Schiffn.	Macaronesian endemic	Near threatened
	*Isopaches bicrenatus* (Schmidel ex Hoffm.) H.Buch	Native	Least concern
	*Jubula hutchinsiae* (Hook.) Dumort.	Native	Least concern
	*Lejeunea cavifolia* (Ehrh.) Lindb.	Native	Least concern
	*Lejeunea ecklonian*a Lindenb.	Native	Least concern
	*Lejeunea flava* (Sw.) Nees	Native	Near threatened
	*Lejeunea hibernica* Bischl., H.A.Mill. & Bonner ex Grolle	European endemic	Near threatened
	*Lejeunea lamacerina* (Steph.) Schiffn.	Native	Least concern
	*Lejeunea mandonii* (Steph.) Müll.Frib.	Native	Vulnerable
	*Lejeunea patens* Lindb.	Native	Least concern
	*Lepidozia reptans* (L.) Dumort.	Native	Least concern
	*Leptoscyphus cuneifolius* (Hook.) Mitt.	Native	Least concern
	Leptoscyphus porphyrius subsp. azoricus (H.Buch & Perss.) Vanderp. & Heinrichs	Azorean endemic	Endangered
	*Lophocolea bidentata* (L.) Dumort.	Native	Least concern
	*Lophocolea fragrans* (Moris & De Not.) Gottsche, Lindenb. & Nees	Native	Least concern
	*Marchesinia mackaii* (Hook.) Gray	European endemic	Least concern
	*Metzgeria furcata* (L.) Corda	Native	Least concern
	*Microlejeunea ulicina* (Taylor) Steph.	Native	Least concern
	*Myriocoleopsis minutissima* (Sm.) R.L.Zhu, Y.Yu & Pócs	Native	Least concern
	*Nowellia curvifolia* (Dicks.) Mitt.	Native	Least concern
	*Plagiochila bifaria* (Sw.) Lindenb.	Native	Least concern
	*Porella canariensis* (F.Weber) Underw.	European endemic	Least concern
	*Porella obtusata* (Taylor) Trevis.	Native	Least concern
	*Radula aquilegia* (Hook.f. & Taylor) Gottsche, Lindenb. & Nees	European endemic	Least concern
	*Radula carringtonii* J.B.Jack	European endemic	Near threatened
	*Radula holtii* Spruce	European endemic	Near threatened
	*Radula wichurae* Steph.	Macaronesian endemic	Near threatened
	*Riccardia chamedryfolia* (With.) Grolle	Native	Least concern
	*Saccogyna viticulosa* (L.) Dumort.	European endemic	Least concern
	*Scapania gracilis* Lindb.	Native	Least concern
	*Scapania nemorea* (L.) Grolle	Native	Least concern
	*Telaranea azorica* (H.Buch & Perss.) Pócs	Macaronesian endemic	Near threatened
	*Telaranea europaea* J.J.Engel & G.L.Merr.	Native	Least concern
